# Reproductive Impacts of African Animal Trypanosomiasis in West African Dwarf Goats—Mechanistic Insights into Trypanotolerance Survival–Fertility Trade-Off: A Systematic Review

**DOI:** 10.3390/vetsci13060535

**Published:** 2026-05-29

**Authors:** Ugochinyere J. Njoga, Emmanuel O. Njoga, Izuchukwu S. Ochiogu, John I. Ihedioha, James W. Oguttu

**Affiliations:** 1 Department of Veterinary Obstetrics and Reproductive Diseases, Faculty of Veterinary Medicine, University of Nigeria, Nsukka 410001, Nigeria; izuchukwu.ochiogu@unn.edu.ng; 2 Department of Agriculture and Animal Health, College of Agriculture and Environmental Sciences, University of South Africa, Florida Science Campus, Roodepoort, Johannesburg 1709, South Africa; joguttu@unisa.ac.za; 3 Department of Veterinary Public Health and Preventive Medicine, Faculty of Veterinary Medicine, University of Nigeria, Nsukka 410001, Nigeria; 4 Department of Veterinary Pathology, Faculty of Veterinary Medicine, University of Nigeria, Nsukka 410001, Nigeria; john.ihedioha@unn.edu.ng

**Keywords:** follicular atresia, hypothalamic–pituitary–gonadal axis, oxidative stress, reproductive dysfunction, trypanosome infections, West African Dwarf goats

## Abstract

Trypanosomiasis remains a major constraint to small ruminant production in sub-Saharan Africa, particularly among smallholder farmers. Although WAD goats are considered trypanotolerant because of their relatively low mortality during chronic infection, recent findings show that this survival is at the expense of reproductive efficiency. To back up this claim with scientific evidence, this review followed PRISMA guidelines and systematically searched PubMed, Scopus, and Web of Science for important studies published between January 1980 and February 2026. Search terms included African animal trypanosomiasis, *Trypanosoma* spp., WAD goats, reproductive dysfunction, trypanotolerance, oxidative stress, and hypothalamic–pituitary–gonadal axis. Of the 1245 retrieved articles, 14 met the inclusion criteria. Evidence from the included studies indicates that chronic trypanosome infection disrupts reproduction through interconnected mechanisms involving systemic inflammation, oxidative stress, endocrine imbalance, and impaired gonadal function. Available evidence suggests that *T. brucei* is frequently associated with ovarian dysfunction and embryonic loss, whereas *T. congolense* has been linked in some studies to uterine pathology and gestational reproductive disturbances. Female goats commonly exhibit irregular oestrous cycles, embryonic loss, and prolonged kidding intervals, while males develop impaired spermatogenesis, abnormal sperm morphology, and reduced testosterone levels. These reproductive impairments reduce kid output, milk yield, herd productivity, and household livelihood resilience. Integrated control strategies combining vector control, targeted chemotherapy, nutritional support, and selective breeding are essential for preserving both fertility and survival in trypanosome-endemic areas.

## Simple Summary

African animal trypanosomiasis (AAT) is a significant but often under-recognised constraint to small ruminant productivity, particularly in West African Dwarf (WAD) goats. Although WAD goats survive chronic infections due to trypanotolerance, this often occurs at the expense of reproductive efficiency. Evidence from 14 systematically selected studies shows that trypanosome infections disrupt reproduction through inflammation, oxidative stress, and endocrine imbalances, impairing ovarian and testicular function. *Trypanosoma brucei* may primarily affect ovarian function and embryonic survival, whereas *Trypanosoma congolense* is linked to uterine pathology and abortions in mid-to-late gestation. Affected females show irregular oestrous cycles, extended inter-kidding interval, reproductive hormonal abnormalities and pregnancy losses, while males exhibit reduced sperm quality and testosterone levels. These subclinical reproductive deficits reduce kid output, milk yield, and the overall herd productivity. Integrated strategies combining vector control, chemotherapy, nutrition, and selective breeding are critical to maintaining both survival and fertility in WAD goats in trypanosome endemic regions.

## 1. Introduction

Goat production is central to rural livelihoods across sub-Saharan Africa, where over 95% of the world’s goats are raised under low-input livestock production systems [1]. In humid and sub-humid West and Central Africa, the WAD goat is one of the most widely distributed indigenous breeds. Its small body size, early maturity, relatively high prolificacy, and ability to thrive under village management systems make it particularly valuable to resource-poor households [2]. Unlike cattle, WAD goats require lower capital investment, reproduce faster, and serve as liquid financial assets that can be sold during emergencies. They provide meat, limited quantities of milk, manure, and socio-cultural value, contributing significantly to food security and poverty alleviation in rural communities. Reproductive efficiency underpins the economic viability of WAD goat production, with short kidding intervals, high conception rates, and strong kid survival, enhancing flock growth and annual off-take [3]. Even modest reductions in fertility parameters can substantially reduce household income and animal-source protein availability. Thus, reproductive prolificacy, not merely survival or disease tolerance, is the most meaningful biological determinant of productivity in smallholder goat systems.

Animal trypanosomiasis remains one of the most important vector-borne diseases limiting livestock productivity across approximately 10 million km^2^ of sub-Saharan Africa [4,5,6]. The disease is caused primarily by *Trypanosoma congolense*, *Trypanosoma brucei brucei,* and *Trypanosoma vivax*, which are transmitted cyclically by tsetse flies (*Glossina* spp.), and *Trypanosoma evansi*, which is mechanically transmitted by biting flies, such as tabanids or *Stomoxys*, allowing the parasite to extend beyond traditional tsetse belts [7]. The AAT has historically been studied primarily in cattle due to its dramatic clinical presentation and economic losses, but small ruminants are now increasingly being recognised as both affected hosts and epidemiological reservoirs [8]. In goats, AAT often manifests as chronic infection characterised by intermittent parasitaemia, anaemia, weight loss, and reduced productivity rather than high mortality [9]. Because death rates in WAD goats are generally lower than in susceptible exotic breeds, the impact of AAT in this species has often been considered limited due to inherent trypanotolerance in WAD goats. This perception has shaped both research priorities and policy narratives, leading to trypanotolerance being overemphasised.

The economic burden of AAT remains substantial. It is estimated that the disease threatens livestock production across more than 10 million km^2^ of sub-Saharan Africa, exposing approximately 50 million cattle and millions of small ruminants to infection risk annually [5,10]. Production losses associated with mortality, reduced fertility, decreased milk yield, and impaired growth have been estimated to exceed USD 4–5 billion per year in affected regions [11,12]. While most economic assessments focus on cattle, the contribution of reproductive losses in small ruminants remains poorly quantified.

### Historical Narrative of Trypanotolerance

The WAD goat is widely described as “trypanotolerant,” denoting the capacity of certain indigenous breeds to survive and remain productive under trypanosome challenge, typically with lower parasitaemia and milder anaemia than susceptible breeds [13]. This concept arose from early observations that indigenous African livestock could endure chronic infection while maintaining relatively stable haematological indices compared with exotic breeds [13]. However, these early studies focused largely on survival and parasitaemia control, with limited attention to reproductive outcomes.

In ruminants and ungulates generally, the WAD goat is regarded as one of the most trypanotolerant breeds [13]. While trypanotolerance has been extensively characterised in cattle such as N’Dama and Muturu, where genetic mechanisms modulate parasitaemia and anaemia severity, similar resilience is often inferred for WAD goats [13,14]. More importantly, trypanotolerance does not imply complete resistance or preservation of optimal productivity. Its definition primarily centres on survival, reduced clinical severity, and haematological stability, with far less emphasis on reproductive function. Emerging evidence indicates that even in trypanotolerant goats, chronic infection disrupts progesterone dynamics, impairs oestrous cyclicity, and increases reproductive losses [15]. Thus, while survival is maintained, it usually occurs at the cost of compromised fertility and reproductive efficiency.

This review advances the hypothesis that trypanotolerance in WAD goats represents a biological trade-off, involving preservation of life at the expense of optimal reproduction and herd productivity. Chronic immune activation, anaemia, oxidative stress, and inflammatory cytokine release during AAT may subtly disrupt the hypothalamic–pituitary–gonadal axis, impair ovarian steroidogenesis, and reduce luteal competence. The result is subfertility rather than overt sterility, manifested as irregular cycles, silent oestrus, reduced conception rates, early embryonic loss, decreased milk yield, and prolonged kidding intervals. Such subclinical reproductive disruptions have profound implications for smallholder productivity. In low-input systems, even small declines in fertility can significantly reduce annual flock output and household income. Therefore, focusing solely on mortality underestimates the true burden of AAT in trypanotolerant goats.

## 2. Literature Search Strategy and Selection Criteria

This review followed the Preferred Reporting Items for Systematic Reviews and Meta-Analyses (PRISMA) guidelines and checklist [16,17] to ensure transparency, reproducibility, and methodological rigour while minimising bias. Relevant studies on African animal trypanosomiasis and reproductive dysfunction in goats were systematically retrieved, screened for eligibility, and critically appraised. The selection emphasised studies reporting endocrine alterations, ovarian and testicular pathology, and fertility outcomes. The review synthesised mechanistic links between chronic *Trypanosoma* spp. parasitaemia and disruption of reproductive physiology in WAD goats in endemic regions. It examined pathways of ovarian dysfunction, impacts on oestrus synchronisation programmes, and associated herd-level productivity losses. It also evaluated endocrine and reproductive consequences of AAT in WAD goats and considers implications for rural livelihoods. The review further reappraises trypanotolerance as a potential reproductive liability rather than an unequivocal advantage, to inform future research, breeding policy, and disease control strategies in endemic settings.

Electronic database searches were performed in PubMed/MEDLINE, Scopus, the Web of Science Core Collection, and African Journal Online (AJOL). To ensure completeness and minimise publication bias, the reference lists of all eligible articles were manually screened for additional relevant studies. Search strategies combined controlled vocabulary (including MeSH terms where applicable) and free-text keywords linked using Boolean operators (AND/OR). Representative search terms included combinations of “African animal trypanosomiasis,” “*Trypanosoma brucei*,” “*Trypanosoma congolense*,” “*Trypanosoma vivax*,” “prevalence”, “West African Dwarf goats,” “trypanotolerance,” “reproductive dysfunction,” “fertility,” “subfertility,” “hypothalamic–pituitary–gonadal axis,” “progesterone,” “steroidogenesis,” “oxidative stress,” “reactive oxygen species,” “cytokines,” “inflammation,” “follicular atresia,” and “luteal dysfunction.” Search strings were adapted to match the indexing syntax of each database.

Studies published between January 1980 and February 2026 were considered eligible for inclusion. Earlier foundational studies were retained where they provided essential mechanistic insights into endocrine regulation, immunopathology, or the biology of trypanotolerance.

Studies were included if they met the following criteria:

1. They investigated AAT infections in WAD goats.
2. They reported prevalence or examined reproductive outcomes such as oestrous cyclicity, progesterone dynamics, conception rate, abortion, embryonic loss, spermatogenesis, or related endocrine markers.
3. They explored mechanistic pathways involving endocrine disruption, inflammatory mediators, oxidative stress, or molecular signalling processes.
4. They were original research articles, systematic reviews, or authoritative experimental studies published in peer-reviewed journals in English.

Studies were excluded if they met any of the following criteria:

1. They focused exclusively on non-reproductive clinical manifestations without relevance to epidemiology, fertility or endocrine function.
2. They were conducted solely in non-ruminant species without mechanistic applicability to goats.
3. They were conference abstracts lacking full methodological details.
4. They lacked sufficient methodological rigour or clearly defined prevalence or reproductive outcome measures.

**Figure 1:**
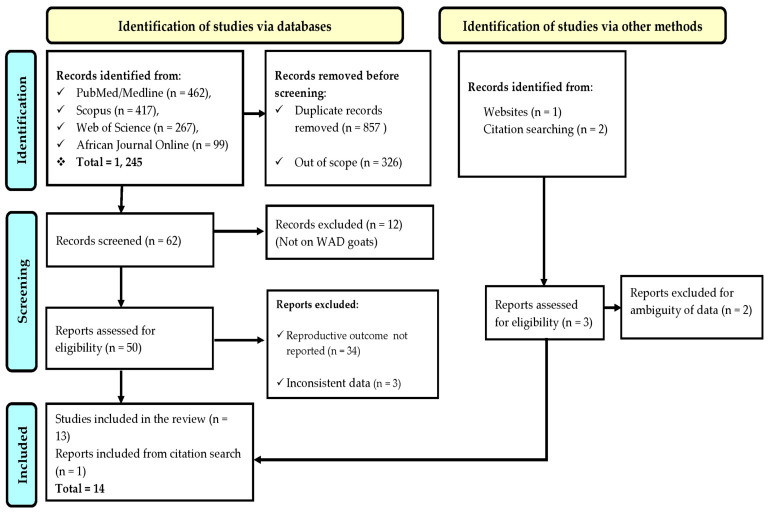
PRISMA flow diagram illustrating the identification, screening, eligibility assessment, and inclusion of studies examining African animal trypanosomiasis and reproductive dysfunction in WAD goats.

Following literature retrieval, records were exported to reference management software, and duplicates were removed. Titles and abstracts were screened for relevance, after which full texts of potentially eligible studies were assessed. Reasons for exclusion were as outlined in the exclusion criteria above. The study selection process is summarised in a PRISMA flow diagram (Figure 1). A total of 1245 records were retrieved. After removal of duplicates (*n* = 857) and out of scope (*n* = 326), 62 titles and abstracts were screened. Of these, 50 full-text articles were assessed, and only 14 met the inclusion criteria. Since goat-specific reproductive studies on trypanosomosis were limited, mechanistic interpretation was cautiously supplemented using comparative reproductive, endocrinological, and immunopathological literature from related ruminant and mammalian models. Such references were used to contextualise biological plausibility and potential mechanistic pathways rather than as primary evidence within the PRISMA-derived synthesis. A simplified methodological quality assessment of included studies was conducted based on study design, reporting clarity, and sample size categories to support interpretation of findings. The risk of bias table (Table S1) and PRISMA 2020 checklist (Table S2) are presented as Supplementary Materials.

## 3. Results

This systematic review synthesised evidence from 14 PRISMA-eligible studies investigating the reproductive consequences of trypanosomosis in WAD goats (Table 1). Collectively, the studies associated AAT with irregular oestrous cycles, reduced conception rates, prolonged inter-kidding intervals, embryonic loss, abortion, impaired spermatogenesis, and reduced reproductive efficiency. Experimental infections involving *Trypanosoma congolense*, *T. vivax*, and *T. brucei* demonstrated altered progesterone concentrations, disrupted luteinising hormone (LH) profiles, ovarian pathology, and testicular degeneration in infected goats. Evidence directly derived from WAD goat studies was strongest for endocrine disruption, altered cyclicity, reduced fertility, and reproductive wastage, while mechanistic interpretations regarding endocrine disruption and gonadal pathology were supplemented by comparative evidence from related ruminants and other mammalian studies. Molecular diagnostic methods consistently detected higher trypanosome prevalence rates than microscopy-based approaches, suggesting that subclinical infections and associated reproductive losses may be underestimated in endemic production systems. Overall, although WAD goats exhibit relative trypanotolerance for survival, chronic trypanosome infection may still compromise reproductive performance and herd productivity.

## 4. Overview of Species-Specific Trypanosome-Induced Reproductive and Endocrine Disruptions in Goats

Table 2 synthesises the reproductive, endocrine, and molecular effects of major African trypanosome species in goats, revealing consistent patterns of fertility impairment across species and host categories. *T. brucei brucei* and *T. congolense* predominantly disrupt female reproductive function through suppression of progesterone, LH, and oestradiol, leading to irregular cycles, anoestrus, and embryonic loss, largely mediated by hypothalamic–pituitary–gonadal axis dysfunction and inflammatory pathways [18,19,20]. In contrast, *T. vivax* and *T. evansi* exerted pronounced effects on male fertility, inducing testicular degeneration, reduced testosterone, and poor semen quality [21,22,23,24]. Notably, *T. vivax* also contributed to abortion and foetal loss via placental damage [24,25]. Across all species, mechanisms such as oxidative stress, chronic inflammation, and endocrine dysregulation converge to produce subfertility at both individual and herd levels (Table 2). These findings emphasise that reproductive compromise is a central, yet often underappreciated, outcome of caprine trypanosomiasis.

### 4.1. Major African Trypanosome Species Affecting Goats

African animal trypanosomiasis in small ruminants is caused by several haemoflagellate protozoa of the genus *Trypanosoma*, traditionally divided into tsetse-transmitted (cyclical) and mechanically transmitted species. In West and Central Africa, the classical tsetse-transmitted pathogens include *Trypanosoma congolense*, *Trypanosoma vivax*, and *Trypanosoma brucei brucei*. These parasites undergo developmental cycles within *Glossina* spp. (Tsetse flies) before transmission to mammalian hosts and are responsible for the syndrome historically termed “nagana” [6,28].

Among these species, *T. congolense* is widely regarded as the most pathogenic in ruminants, frequently inducing chronic anaemia, immunopathology, and reproduction losses. The *T. vivax*, in contrast, can produce acute parasitaemic episodes with marked systemic inflammatory responses but is also capable of establishing chronic infections. *T. brucei brucei* infections in goats are less commonly documented but may result in severe systemic disease under experimental or heavy natural challenge conditions [29,30].

More importantly, *T. evansi* must also be considered in a comprehensive discussion of trypanosomes affecting goats in Africa. Although *T. evansi* evolved from *T. brucei* and lost the capacity to complete cyclical development in the tsetse fly, it is efficiently transmitted mechanically by hematophagous flies such as *Tabanids* and *Stomoxys* species [29,30]. This transmission flexibility allows *T. evansi* to extend beyond traditional tsetse belts, and it is responsible for surra, a disease of major veterinary significance in Africa and beyond.

While *T. evansi* is more frequently associated with camels, horses, and cattle, infections in goats are documented and may be underdiagnosed due to nonspecific clinical presentation [31]. Like classical AAT species, *T. evansi* induces anaemia, systemic inflammation, and metabolic disturbances that can compromise reproductive performance [29,30]. Therefore, despite its non-cyclical transmission, it belongs within the broader pathogenic complex affecting small ruminant reproduction in endemic regions. Mixed infections involving two or more trypanosome species are common under field conditions, particularly where vector densities are high and diagnostic sensitivity is limited [26,27,32]. Such co-infections may exacerbate inflammatory and endocrine disturbances, potentially amplifying reproductive consequences.

For WAD goats, the biological distinction between cyclical and mechanical transmission is epidemiologically important but pathophysiologically secondary. Regardless of vector, these parasites share core mechanisms: antigenic variation via variant surface glycoproteins, recurrent parasitaemia, chronic immune activation, and progressive anaemia. These mechanisms are central to understanding how apparently “tolerant” goats may survive infection while experiencing subtle yet significant reproductive endocrine disruption. Thus, a comprehensive evaluation of the trypanotolerance paradox must encompass both tsetse-transmitted AAT species and mechanically transmitted *T. evansi*, particularly when examining impacts on progesterone dynamics and oestrous cyclicity. Table 3 below compares the main African trypanosome species affecting WAD goats, highlighting their transmission modes, parasitaemia patterns, hormonal disruptions, and relative reproductive impacts.

### 4.2. Epidemiology of African Animal Trypanosomiasis in Goats

African animal trypanosomiasis remains enzootic across much of sub-Saharan Africa, with goats, particularly WAD goats, serving as both resilient hosts and under-recognised reservoirs. The epidemiology of AAT in goats is shaped by vector ecology (notably *Glossina* spp.), host genetics, management systems, and diagnostic sensitivity. In humid and sub-humid zones of West and Central Africa, where WAD goats predominate, persistent tsetse challenge drives endemic stability characterised by chronic, often subclinical infections with low parasitaemia.

Small ruminants are frequently underrepresented in trypanosomiasis surveillance programs, yet field studies demonstrate substantial exposure rates in endemic regions. Prevalence in goats across West Africa commonly ranges from 5% to 40%, depending on season, diagnostic method, and ecological zone [36]. Molecular diagnostics consistently reveal higher prevalence than microscopy, indicating that subclinical and low-parasitaemia infections are common. The WAD goats are typically managed under extensive or semi-extensive systems, grazing in riverine and forest margins where tsetse density is high. Seasonal peaks in vector abundance during rainy periods correspond to increased transmission intensity [1], while chronic infections often persist through dry seasons, maintaining a reservoir for subsequent transmission cycles.

Prevalence of small ruminant trypanosomiasis varies widely across African settings, reflecting diagnostic method sensitivity and ecological differences. Molecular assays (e.g., PCR) consistently reveal higher infection rates in goats (up to 17.5% in Somalia) and sheep (up to 21% in Gabon) compared with traditional parasitological or microscopy approaches in Nigeria reporting < 10% prevalence (Table 4). Pooled molecular data from Uganda further confirm moderate prevalence in goats and sheep (Table 4). These findings highlight the substantial underestimation of infection burden when relying solely on conventional microscopy, particularly in trypanotolerant breeds where parasitaemia remains intermittently detectable [37,38,39].

In West African settings, parasitological surveys often report lower apparent prevalence (1–7%) in goats [40,41], but these figures likely underestimate true infection rates due to diagnostic limitations. The predominant species infecting goats include *Trypanosoma vivax*, *T. congolense*, and *T. brucei*, with *T. vivax* frequently dominant in areas of reduced tsetse density owing to its capacity for mechanical transmission by hematophagous flies [43]. This expands the epidemiological range of AAT beyond classical tsetse belts into peri-urban and mixed farming systems.

The trypanotolerance of WAD goats significantly modifies AAT epidemiology. These animals typically maintain controlled parasitaemia, reduced anaemia severity, and enhanced survival under infection pressure. However, this adaptive advantage facilitates prolonged pathogen persistence, allowing goats to function as reservoirs that sustain transmission within multi-host systems. Because goats are less intensively monitored than cattle, reproductive losses linked to trypanosome exposure frequently go undocumented. Abortions, extended inter-kidding intervals, and reduced conception rates are often misattributed to nutritional or managerial deficiencies rather than blood parasitic infection, contributing to systematic underestimation of the reproductive burden of AAT. Overall, the epidemiology of AAT in goats reflects a complex interplay between vector dynamics, host adaptation, and diagnostic limitations. In WAD goats, trypanotolerance underpins survival in endemic environments but may inadvertently promote chronic infection persistence, reinforcing a survival–fertility trade-off with significant implications for reproductive performance and herd productivity.

## 5. Trypanosome Infection Dynamics and Systemic Pathophysiology

Following inoculation by the tsetse fly, trypanosomes proliferate in the blood and lymphatic systems, evading host immunity through variant surface glycoprotein (VSG) switching. This antigenic variation drives cyclical parasitaemia and sustained immune activation [44,45]. The host response involves pro-inflammatory cytokines—including tumour necrosis factor-α (TNF-α) and interferon-γ (IFN-γ), which contribute to anaemia, cachexia, and metabolic dysregulation [44,45]. Chronic anaemia reduces tissue oxygenation, including in ovarian tissue. Hypoxia and inflammatory mediators impair granulosa cell function and steroidogenesis, potentially reducing progesterone synthesis. Experimental infections in WAD goats demonstrate altered plasma progesterone concentrations and disrupted luteal function [46]. These endocrine perturbations occur even when animals survive infection and maintain moderate body condition.

Additionally, trypanosome-induced oxidative stress and immune-mediated haemolysis may impair hypothalamic–pituitary signalling. Cytokine-mediated suppression of gonadotropin-releasing hormone (GnRH) and LH pulses has been observed in other chronic inflammatory states and is biologically plausible in *Trypanosoma* infections [47,48]. Thus, the infection dynamics of AAT create a sustained inflammatory–metabolic environment incompatible with optimal reproductive hormone cycling. Survival under such conditions does not imply preserved fertility.

### Trypanosome Infection Biology Matters for Oestrous Synchronisation

Progesterone-based oestrus synchronisation protocols rely on predictable luteal regression, synchronised follicular waves, and adequate progesterone priming [49]. Chronic trypanosome infection may compromise each of these steps. Reduced endogenous progesterone, altered LH pulsatility, and impaired folliculogenesis may decrease synchronisation success, lower conception rates following artificial insemination or natural mating, and increase early embryonic mortality [18,50]. In endemic settings, failure of synchronisation programs is often attributed to handling stress or nutrition. However, underlying parasitaemia may represent a hidden confounder. Understanding infection dynamics is therefore essential not only for natural fertility but also for assisted reproductive technologies increasingly promoted in smallholder goat improvement schemes.

## 6. Pathophysiological Mechanisms of Reproductive Endocrine Disruption in Trypanosome-Infected WAD Goats

### 6.1. Disruption of the Hypothalamic–Pituitary–Gonadal Axis

Normal reproductive cyclicity in goats depends on tightly coordinated signalling within the hypothalamic–pituitary–gonadal (HPG) axis. Pulsatile secretion of GnRH from the hypothalamus stimulates LH and follicle-stimulating hormone (FSH) release from the anterior pituitary, which in turn regulates folliculogenesis, ovulation, and corpus luteum (CL) function. Progesterone produced by the CL provides negative feedback essential for cycle regulation and pregnancy maintenance. *Trypanosoma* infections in animals generate chronic systemic inflammation characterised by elevated pro-inflammatory cytokines such as TNF-α, IFN-γ and interleukin-1β (IL-1β) [50,51]. These mediators can directly suppress hypothalamic GnRH secretion and alter pituitary responsiveness [52]. Although specific neuroendocrine studies in goats remain limited, analogous inflammatory suppression of the HPG axis has been documented across mammalian species experiencing chronic infection or immune activation.

**Figure 2:**
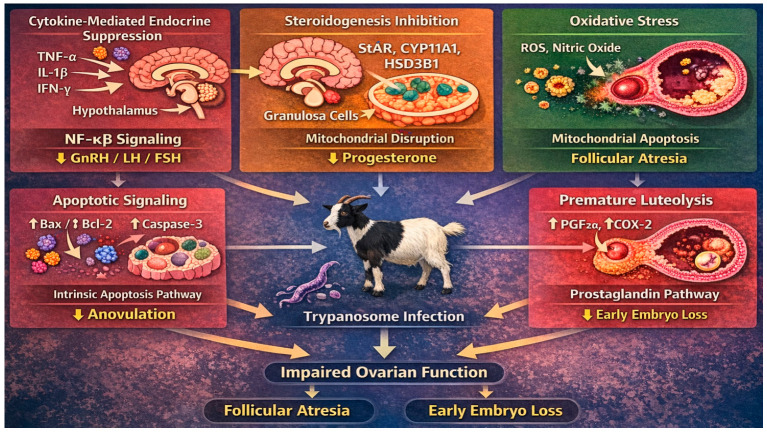
Mechanistic pathways linking African animal trypanosomiasis to reproductive dysfunction in West African Dwarf goats.

In experimental infections of West African Dwarf goats with *Trypanosoma congolense*, altered plasma luteinising hormone concentrations and disrupted oestrous cycles have been documented [20]. The reduction in LH pulsatility compromises ovulatory timing and follicular maturation. These endocrine perturbations occur even when clinical signs are mild, supporting the concept that trypanotolerance does not equate to preserved reproductive physiology. Furthermore, chronic parasitaemia leads to metabolic stress and negative energy balance, both known suppressors of GnRH secretion. The combined effects of immune-mediated and metabolic suppression place the reproductive axis in a state of functional inhibition, predisposing to irregular oestrous cycles, silent heat, and subfertility. The complex interactions between chronic trypanosome infection, systemic inflammation, oxidative stress, and HPG axis disruption, and their effects on goat reproductive function, are summarised in Figure 2.

Chronic infection with *Trypanosoma* species induces systemic inflammation and oxidative stress, disrupting the hypothalamic–pituitary–gonadal (HPG) axis. This leads to impaired ovarian and testicular steroidogenesis, follicular atresia, luteal insufficiency, gametogenic apoptosis, and reproductive outcomes including irregular oestrous cycles, early embryonic loss, mid-to-late gestational abortions, impaired spermatogenesis, and reduced libido. *T. brucei* is primarily associated with ovarian dysfunction and embryonic loss, whereas *T. congolense* is more strongly linked to uterine pathology. Arrows indicate the flow of mechanistic effects, highlighting the trade-off between survival and fertility in WAD goats.

### 6.2. Ovarian Steroidogenesis and Progesterone Insufficiency

Progesterone is central to oestrous cycle regulation and early pregnancy maintenance. In goats, adequate luteal progesterone concentrations are required for uterine receptivity, embryo implantation, and suppression of premature luteolysis [53,54,55]. Any reduction in progesterone synthesis or luteal competence can significantly reduce conception rates [53]. Experimental evidence demonstrates that trypanosome-infected WAD goats exhibit altered plasma progesterone profiles [20]. In a controlled study, infection with *Trypanosoma congolense* resulted in significantly reduced progesterone concentrations during the luteal phase compared to uninfected controls [20]. This suggests impaired corpus luteum function, likely secondary to inflammatory and hypoxic mechanisms.

Anaemia, the hallmark of AAT, reduces oxygen delivery to ovarian tissue. The ovary is highly vascularised and metabolically active; hypoxia impairs granulosa and luteal cell steroidogenic enzyme activity, including cholesterol side-chain cleavage enzyme (P450scc) and 3β-hydroxysteroid dehydrogenase [56]. Reduced enzymatic efficiency compromises progesterone synthesis. Additionally, systemic cytokines such as TNF-α can directly inhibit luteal steroidogenesis [56]. Experimental studies in ruminants show that inflammatory mediators promote luteolytic pathways and increase prostaglandin F2_α_ production, accelerating CL regression [57,58]. In infected goats, this may translate into shortened luteal phases and premature return to oestrus. Such endocrine instability has direct implications for progesterone-based oestrus synchronisation programs, which depend on predictable luteal regression and uniform follicular wave emergence. In chronically infected goats, endogenous progesterone insufficiency may reduce synchronisation success and post-mating conception rates.

### 6.3. Follicular Dynamics, Ovulatory Failure, and Ovarian Pathology

Beyond luteal dysfunction, trypanosomes also influence follicular development. Chronic inflammation and oxidative stress promote follicular atresia by inducing apoptosis in granulosa cells [59]. Reactive oxygen species (ROS) generated during persistent immune activation damage cellular membranes and mitochondrial integrity, compromising follicular viability [60]. Histopathological studies in experimentally infected small ruminants reveal ovarian congestion, degeneration of developing follicles, and reduced numbers of mature Graafian follicles [61]. These structural changes correspond with clinical observations of anoestrus and irregular oestrous behaviour. Subclinical ovulatory failure may occur even when behavioural oestrus is observed. Moreover, infection with *Trypanosoma vivax* has been associated with reproductive wastage, including early embryonic loss and abortion in small ruminants, particularly under high parasitaemic burdens [62]. Embryonic loss may result from inadequate progesterone support, uterine inflammatory responses, or systemic febrile episodes disrupting early gestation. Collectively, these ovarian-level disturbances reinforce the central thesis of this review: survival under trypanosome challenge does not guarantee preserved fertility. Instead, chronic infection establishes a biologically stressful environment that progressively undermines reproductive efficiency. These reproductive disturbances translate into measurable reductions in herd productivity and reproductive output in affected goat populations. A summary of the documented reproductive and productivity consequences of African animal trypanosomiasis in West African Dwarf goats is presented in Table 5.

### 6.4. Biological Basis of the Trypanotolerance Paradox

The concept of trypanotolerance has historically emphasised haematological resilience and survival advantage. However, immune containment of parasitaemia requires sustained inflammatory activation. This chronic immune engagement diverts metabolic resources and exposes endocrine tissues to cytokine-mediated disruption. Thus, the paradox emerges: the same adaptive responses that permit survival may compromise reproductive endocrine stability. In WAD goats, the cost of tolerance may manifest not as mortality but as subfertility, characterised by prolonged kidding intervals, reduced conception rates, silent oestrus, and increased embryonic loss [64]. Recognising this trade-off shifts evaluation metrics from survival-based to productivity-based outcomes. For smallholder systems dependent on rapid flock turnover, reproductive efficiency is the true currency of resilience.

### 6.5. Effects of Trypanosome Infection on Oestrous Cycle Characteristics and Progesterone-Based Synchronisation Protocols in West African Dwarf Goats

Progesterone-based oestrus synchronisation is a central component of reproductive management in small ruminants, which enables controlled breeding, fixed-time artificial insemination, and improved kidding efficiency [61]. Exogenous progesterone delivered via intra-vaginal devices such as Controlled Internal Drug Release inserts or fluorogestone acetate sponges mimics the luteal phase by suppressing oestrus and ovulation [61,62]. Following withdrawal, the decline in progesterone initiates follicular maturation, a luteinising hormone surge, and synchronised oestrus [61,62]. The success of these protocols depends on a functional hypothalamic–pituitary–gonadal axis, adequate follicular reserves, proper corpus luteum activity, and stable metabolic conditions. Under optimal circumstances, oestrus response rates can exceed 85% and conception rates surpass 70%, particularly when equine chorionic gonadotrophin is included to stimulate follicular growth [65,66].

Chronic trypanosome infection disrupts these physiological requirements at multiple levels, thereby impairing oestrous cyclicity and synchronisation efficiency. Suppression of gonadotrophin-releasing hormone secretion, irregular luteinising hormone pulsatility, and compromised ovarian steroidogenesis collectively destabilise the endocrine environment required for normal cycle progression. Studies in West African Dwarf goats have reported prolonged or irregular inter-oestrus intervals, often associated with luteal insufficiency or silent oestrus, alongside weak behavioural signs that hinder detection and mating success [20,34]. In addition, cytokine-mediated upregulation of prostaglandin F2_α_ contributes to premature luteal regression, while anovulatory cycles are common in chronically infected animals with moderate to high parasitaemia [58]. These disruptions significantly reduce the proportion of animals exhibiting oestrus within the expected synchronisation window.

The pharmacodynamics of exogenous progesterone could be further compromised by infection-induced systemic alterations. Hepatic dysfunction, anaemia, and inflammation can modify hormone metabolism, resulting in reduced circulating progesterone levels and inconsistent endocrine responses [67,68]. Oxidative stress and immune-mediated ovarian damage impair corpus luteum responsiveness, weakening the feedback mechanisms necessary for synchronisation [67]. Furthermore, increased follicular atresia and reduced follicular dominance limit the ovulation of viable oocytes following progesterone withdrawal [67]. These combined effects culminate in synchronisation failure, characterised by asynchronous oestrus onset, reduced ovulation rates, and lower conception outcomes.

The reproductive compromise associated with chronic trypanosome infection has significant implications for artificial reproductive technologies and breeding programmes in WAD goats. The effectiveness of fixed-time mating programmes is often reduced, as synchronisation protocols fail more frequently in infected herds, leading to increased labour demands and inefficient resource utilisation [69]. Conception rates following artificial insemination are also diminished, partly due to early embryonic loss arising from inadequate luteal progesterone support [69]. In addition, reproductive wastage is heightened, with increased incidences of early embryonic mortality and silent oestrus contributing to reduced kidding output per breeding season [69]. These challenges could translate into measurable economic losses, such as extended generation intervals, slower flock expansion, and reduced household income from goat production systems.

**Figure 3:**
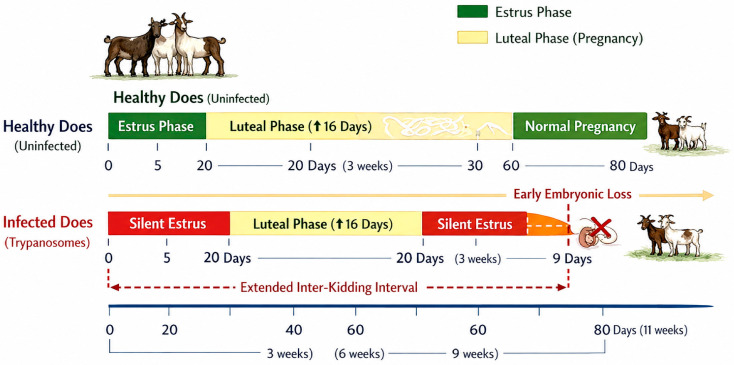
Reproductive Timeline Showing Oestrous Cycle Disruption in Infected versus Uninfected WAD Goats.

Mitigating synchronisation failure in trypanosome-endemic regions requires an integrated and context-specific management approach. Pre-synchronisation screening for parasitaemia is essential, as early detection and treatment of subclinical infections can minimise endocrine disruption and improve reproductive outcomes. Strategic administration of trypanocidal agents prior to progesterone device insertion may reduce infection-related inflammatory and endocrine disturbances, which could improve luteal function and oestrous expression in infected animals [70]. Nutritional support, particularly through antioxidant supplementation, plays a complementary role by improving metabolic stability and reducing oxidative damage to ovarian tissues, thereby enhancing follicular responsiveness [67,68]. Furthermore, modifications to conventional synchronisation protocols, such as prolonged progesterone exposure or the inclusion of equine chorionic gonadotropin, may partially compensate for impaired follicular dynamics and improve synchronisation success rates [67,68]. Implementation of these strategies within smallholder systems is feasible but depends on effective extension services, farmer education, and continuous monitoring of both parasitological and reproductive performance indicators. A comparative reproductive timeline, demonstrating how trypanosome infection alters oestrous cycles, extends inter-kidding intervals, and increases early embryonic loss in WAD goats, is illustrated in Figure 3.

## 7. Molecular and Cellular Pathways of Ovarian Dysfunction in Trypanosome-Infected WAD Goats

African *Trypanosoma* infections disrupt ovarian function through a complex interplay of immune-mediated, metabolic, and oxidative mechanisms, ultimately compromising steroidogenesis and follicular viability. At the molecular level, chronic parasitaemia may elicit persistent systemic inflammation characterised by elevated cytokines such as TNF-α, IL-1β, and IFN-γ [71,72,73]. These cytokines may have direct inhibitory effects on ovarian steroidogenic pathways in WAD goats. In granulosa and luteal cells, TNF-α downregulates the expression of key steroidogenic enzymes, including steroidogenic acute regulatory protein (StAR), cytochrome P450 side-chain cleavage enzyme (CYP11A1), 3β-hydroxysteroid dehydrogenase (HSD3B1), and aromatase (CYP19A1), which has been reported in other models [74,75,76]. Suppression of StAR may limit cholesterol transport into mitochondria, the rate-limiting step for progesterone synthesis, while downregulation of CYP11A1 impedes pregnenolone production [77]. Together, these molecular perturbations reduce luteal progesterone output, shorten the luteal phase, and destabilise the oestrous cycle.

Concurrent oxidative stress amplifies ovarian injury. Trypanosome infections induce ROS production both via host macrophage NADPH oxidase activity and through parasite metabolic by-products [78]. Elevated ROS levels may trigger lipid peroxidation in granulosa and theca cell membranes, damage mitochondrial integrity, and activate apoptotic pathways via caspase-3 and caspase-9 [78,79,80]. Additionally, ROS may promote upregulation of pro-apoptotic proteins such as Bax while downregulating anti-apoptotic Bcl-2 in follicular cells, accelerating follicular atresia [81,82,83]. The loss of dominant follicles reduces ovulation rates and contributes to irregular oestrous cycles, anovulation, and silent heat in infected does.

Metabolic disruption further compounds reproductive dysfunction. Chronic infection leads to systemic anaemia and altered hepatic function, reducing cholesterol availability necessary for steroid hormone biosynthesis [84]. Hypocholesterolaemia may constrain substrate availability for CYP11A1-mediated pregnenolone synthesis, while inflammatory signalling through nuclear factor kappa B (NF-κB), which modulates expression of steroidogenic enzymes at the transcriptional level, is biologically plausible [84,85]. Furthermore, pro-inflammatory cytokines stimulate ovarian prostaglandin F2-alpha (PGF2α) synthesis through upregulation of cyclooxygenase-2 (COX-2), promoting premature luteolysis and early regression of the corpus luteum [86,87]. This molecular cascade shortens the luteal phase and destabilises the progesterone-dependent window required for successful conception and early embryo survival.

**Figure 4:**
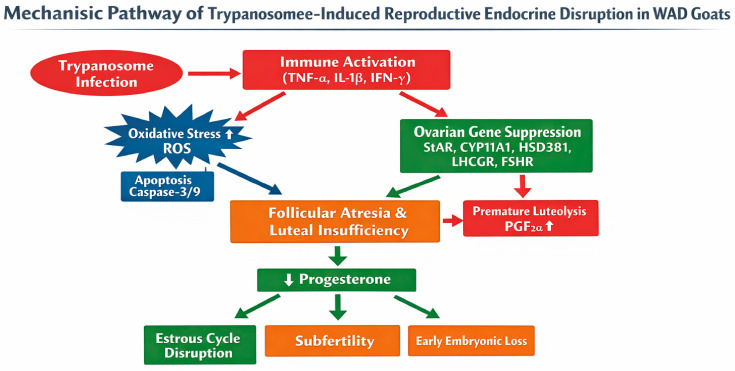
Mechanistic Pathway of Trypanosome-Induced Reproductive Endocrine Disruption in WAD Goats.

Chronic trypanosome infection also interferes with hypothalamic–pituitary signalling. Elevated circulating cytokines and glucocorticoids could reduce hypothalamic GnRH pulse frequency, resulting in decreased pituitary LH and FSH secretion [88,89]. Reduced LH pulsatility diminishes follicular sensitivity to gonadotropins and inhibits the maturation of the dominant follicle. At the gene expression level, downregulation of LH receptor (LHCGR) and FSH receptor (FSHR) in granulosa cells has been observed in chronic inflammatory states and is likely recapitulated in trypanosome-infected goats, compromising ovulatory capacity [90]. Simultaneously, oxidative and inflammatory stress in the ovary induces activation of p53 and other cell-cycle checkpoint proteins, promoting granulosa cell apoptosis and follicular attrition [91,92]. Figure 4 shows the integrated mechanistic pathways by which trypanosome infection disrupts ovarian endocrine function and induces subfertility in WAD goats.

Figure 4 illustrates that chronic trypanosome infection induces persistent parasitaemia and anaemia, triggering systemic inflammatory responses characterised by elevated TNF-α, IFN-γ, and other pro-inflammatory cytokines. These mediators promote mitochondrial dysfunction and excessive generation of reactive oxygen species (ROS), resulting in oxidative stress. Inflammatory and oxidative signals disrupt hypothalamic–pituitary–gonadal (HPG) axis regulation by suppressing GnRH, LH, and FSH secretion. Downstream consequences include impaired ovarian steroidogenesis, reduced progesterone production, granulosa cell apoptosis, follicular atresia, luteal insufficiency, and embryonic loss. Collectively, these mechanisms culminate in subfertility, prolonged inter-kidding intervals, abortion, and reduced herd productivity.

The interplay of immune, oxidative, and metabolic insults also impacts early embryogenesis. Reduced luteal progesterone, elevated PGF2_α_, and inflammatory mediators could create a hostile uterine environment, compromising endometrial receptivity and embryo implantation [93,94]. Genes critical for early embryonic development, such as insulin-like growth factor 1 (IGF1), may be downregulated under chronic inflammatory conditions, further exacerbating reproductive inefficiency [95,96]. Collectively, these molecular disruptions demonstrate that WAD goats’ apparent trypanotolerance at the haematological level comes at a significant reproductive cost, manifesting as subfertility, early embryonic loss, and synchronisation failure. Table 6 below summarises the molecular and cellular mechanisms underlying ovarian dysfunction in trypanosome-infected WAD goats, linking immune, oxidative, and endocrine pathways to reproductive failure.

## 8. Subfertility Manifestations in Trypanosome-Infected West African Dwarf Goats

Chronic African trypanosome infection in WAD goats manifests as a spectrum of reproductive impairments rather than overt mortality. Even in animals demonstrating classical trypanotolerance, subtle disruptions of the hypothalamic–pituitary–gonadal axis, luteal insufficiency, and follicular attrition culminate in observable subfertility, which has profound implications for herd productivity [98]. One of the most consistently reported effects is irregularity of the oestrous cycle [98]. In experimentally infected goats, the interval between oestrus episodes is often prolonged, and behavioural oestrus may be weak or absent, a phenomenon frequently described as silent heat [98]. These alterations are attributable to decreased pulsatile release of LH and FSH, impaired granulosa cell steroidogenesis, and premature corpus luteum regression, all of which compromise follicular dominance and ovulation timing [20,98].

Early embryonic loss is another prominent manifestation. Reduced luteal progesterone output, together with systemic inflammation and oxidative stress, creates a uterine environment unfavourable to implantation and embryonic development [99]. Experimental studies in WAD goats infected with *Trypanosoma congolense* and *T. vivax* demonstrate significantly higher rates of embryo resorption and failed pregnancies compared to uninfected controls [100]. Elevated PGF2_α_ levels, induced by inflammatory cytokines such as TNF-α, contribute to premature luteolysis, further exacerbating early gestational loss [101]. Embryonic mortality may be underreported in field conditions, as abortions can be mistaken for nutritional or environmental failures in low-input systems.

Abortion and perinatal mortality also contribute to reduced reproductive efficiency. While adult mortality in trypanotolerant WAD goats remains low, abortion rates may increase substantially in endemic zones, with perinatal deaths further reducing effective kid output [102]. These losses are associated with both direct parasite effects and secondary metabolic stress. Chronic anaemia, hypoxia, and oxidative damage to reproductive tissues disrupt both oocyte competence and uterine receptivity, producing compounded reproductive inefficiency.

Reduced conception rates following natural or assisted mating are also possible. Goats with subclinical or chronic trypanosome infections frequently fail to conceive despite exhibiting oestrus behaviour, reflecting the integrated effects of luteal insufficiency, follicular atresia, and impaired ovulatory dynamics [103,104]. Such subfertility could be particularly problematic for smallholder systems, where limited herd size amplifies the economic impact of each reproductive failure. In addition, buck fertility may also be compromised. Chronic infection in males can reduce sperm motility, impair spermatogenesis, and alter testosterone levels, further reducing herd fertility potential [62]. Prolonged inter-kidding intervals represent a downstream consequence of these reproductive disruptions. Where healthy WAD does might achieve two breeding cycles per year, trypanosome-infected does often experience extended intervals between successful pregnancies. In village-level management systems, this reduction in kid crop translates directly into fewer animals for household consumption, sale, or reinvestment into the herd. These manifestations illustrate that survival under trypanosome challenge in WAD goats carries a significant reproductive cost.

Subfertility, early embryonic loss, abortion, and extended inter-kidding intervals reduce the effective reproductive output of herds. While haematological tolerance allows adult survival, the hidden cost is diminished productivity, which has cascading effects on income, nutrition, and overall household livelihood security. Recognising these outcomes is essential for framing trypanotolerance not as a purely adaptive trait but as a survival strategy with considerable reproductive trade-offs, providing the rationale for integrated reproductive and parasitic management strategies in endemic regions.

## 9. Herd-Level Productivity Consequences of Trypanosome-Induced Subfertility in WAD Goats

Subfertility resulting from chronic African trypanosome infections translates into measurable reductions in herd-level productivity. In WAD goats, the cumulative effects of irregular oestrous cycles, early embryonic loss, abortions, and extended inter-kidding intervals significantly diminish the effective reproductive output of the herd. Even when mortality is low due to trypanotolerance, these reproductive impairments reduce the number of kids produced per doe per year, constraining herd growth and the potential for incremental income generation [20].

Experimental studies have demonstrated that does infected with *Trypanosoma congolense* or *T. vivax* may experience reductions of up to 30% in conception success and prolonged inter-kidding intervals relative to uninfected controls [20,34]. When extrapolated to field herds, such reductions can substantially delay herd expansion and increase the time required to replace unproductive or aging females. Additionally, reduced ovulation rates and silent oestrus episodes could decrease the predictability and success of both natural mating and fixed-time breeding programs, further amplifying reproductive inefficiency.

Reproductive compromise in males also contributes to herd-level impacts. Chronic infection in bucks can impair spermatogenesis, reduce sperm motility, and decrease circulating testosterone levels, resulting in reduced mating efficiency and lower conception rates at the population level [82,83,84,85,86,87,88,89,90]. The combined effect of subfertility in both sexes increases the proportion of non-productive breeding pairs, creating a bottleneck in herd reproduction that could persist across seasons. Moreover, reduced reproductive output affects the quantity of marketable offspring and milk production. Kids lost due to early embryonic death or abortion represent both immediate nutritional deficits and long-term economic losses. In smallholder systems where WAD goats are a primary source of household income, reduced kidding rates translate into diminished cash flow and limited capacity to purchase feed, veterinary care, or reinvest in herd expansion. These herd-level reproductive losses are often underreported because they occur in the absence of overt clinical signs or mortality, highlighting the hidden cost of trypanotolerance.

**Figure 5:**
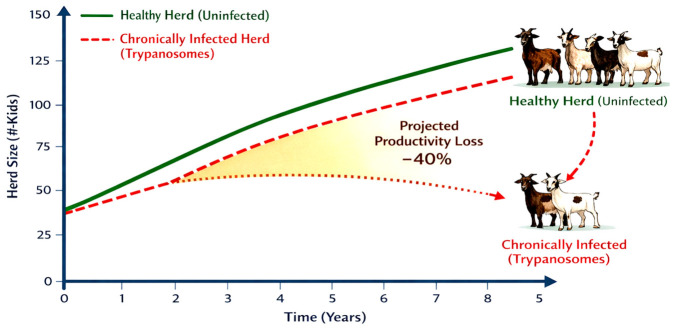
Projected Herd Growth Curves in trypanosome-Infected versus Uninfected WAD Goats.

A study on the economic benefits of interventions against bovine trypanosomiasis in Eastern Africa indicates that herds experiencing chronic trypanosome infection may exhibit a marked reduction in net herd growth per year, depending on infection prevalence, parasitaemia intensity, and management system [29]. In traditional village systems, even small reductions in kid crop have magnified socio-economic consequences due to the reliance on goats for food, income, and social capital. Herd productivity losses are compounded when oestrus synchronisation programs fail due to endocrine disruption, as described in earlier sections, further reducing the efficiency of breeding interventions designed to accelerate herd improvement. Ultimately, the herd-level effects of trypanosome-induced subfertility illustrate a paradox: the survival advantage conferred by trypanotolerance comes at the cost of reduced reproductive efficiency. This hidden cost undermines the resilience of smallholder systems, constraining both biological productivity and economic sustainability. Understanding these dynamics is essential for designing integrated interventions that simultaneously address parasitic control, reproductive management, and rural livelihoods. Figure 5 compares projected herd growth trajectories in uninfected versus chronically trypanosome-infected WAD goats, highlighting the cumulative effect of subfertility on herd expansion.

## 10. Livelihood and Food Security Implications of Trypanosome-Induced Subfertility in WAD Goats

The reproductive inefficiencies induced by African trypanosome infections in WAD goats extend far beyond biological endpoints, with profound implications for rural livelihoods and household food security. In many smallholder farming systems across sub-Saharan Africa, WAD goats are integral to both subsistence and income generation. They provide a steady source of animal protein through meat and milk, generate cash through the sale of offspring, and serve as economic buffers in times of crop failure or financial need [13,28]. When reproductive performance is compromised, these critical functions are undermined.

Subfertility and reproductive losses reduce the annual kid crop, limiting both immediate household nutrition and future herd expansion. In resource-poor settings, where each kid contributes to protein intake, the reduction in available meat can exacerbate dietary protein deficiency. Milk production, while modest in WAD goats, is also affected due to fewer lactating does and lower offspring demand, further decreasing the availability of animal-source protein for children and women, who are typically primary consumers [29].

Economic impacts are equally significant. Reduced kidding rates and increased intervals between pregnancies constrain the saleable output of smallholder herds. Families relying on goat sales for school fees, medical expenses, and other household needs face diminished cash flow. In areas where market access is limited, these reproductive losses translate into prolonged periods of financial vulnerability, reinforcing cycles of poverty. The hidden nature of these losses—occurring without overt mortality—often leads to underestimation by farmers and extension services, resulting in insufficient interventions to address the problem.

Gender and social dimensions further amplify the impact. Women, who frequently manage goat herds in smallholder contexts, bear the brunt of decreased reproductive efficiency. Reduced offspring output limits both household consumption and the ability to sell surplus for income, constraining women’s agency and economic independence. Additionally, youth involved in small ruminant management experience fewer opportunities for skill development, savings, and entrepreneurial activity, perpetuating socio-economic stagnation within rural communities.

From a broader food security perspective, chronic subfertility in WAD goats contributes to systemic nutritional vulnerability. In regions with limited livestock diversity, decreased goat productivity reduces the availability of high-quality animal-source foods, contributing to protein-energy malnutrition and diminished resilience to environmental or economic shocks. The compounded effect of reduced reproductive output and lower kid survival also limits herd replenishment, increasing the risk of long-term herd depletion and sustained livelihood insecurity.

**Figure 6:**
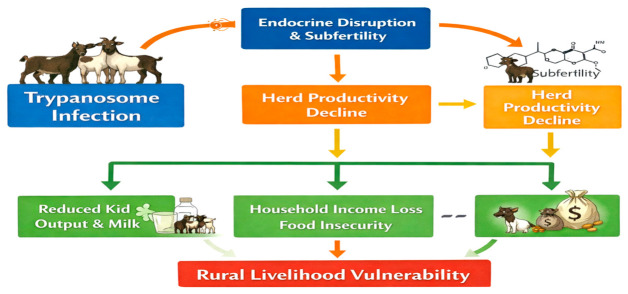
Conceptual Framework Linking Trypanosome Infection, Reproductive Failure, and Rural Livelihood Impacts.

**Figure 7:**
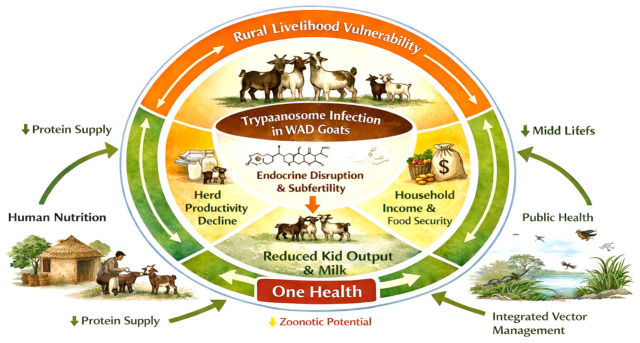
One Health Conceptual Framework Linking Trypanosome Infection in Goats to Rural Nutrition and Livelihood Impacts.

These socio-economic ramifications underscore the central thesis of the trypanotolerance paradox: goats may survive infection, yet the hidden reproductive costs reduce household resilience and nutritional security. Addressing trypanosome-induced subfertility is therefore critical not only for herd productivity but also for safeguarding livelihoods and food security in rural African communities. Integrated interventions, combining parasite control, reproductive management, and nutrition, are essential to mitigate these multidimensional impacts. A conceptual framework linking chronic trypanosome infection to reproductive failure, herd-level productivity losses, and subsequent impacts on rural livelihood vulnerabilities is presented in Figure 6 and Figure 7.

## 11. Integrated Way-Outs: Breaking the Trypanotolerance Paradox

Mitigating the reproductive consequences of chronic African trypanosome infections in WAD goats requires a multi-pronged, integrated approach that addresses parasite control, reproductive management, nutrition, and herd-level productivity. The goal is to preserve fertility while maintaining the survival advantage conferred by trypanotolerance. Pre-breeding parasite screening is fundamental. Detecting subclinical infections through sensitive molecular diagnostics, such as PCR assays targeting species-specific VSG genes, enables timely intervention before oestrus synchronisation programs. Early identification allows strategic trypanocidal therapy with agents such as diminazene aceturate or isometamidium chloride, which reduce parasitaemia, dampen the inflammatory process, and restore endocrine function prior to breeding. Pre-breeding trypanocidal treatment may reduce parasitaemia and associated inflammatory and endocrine disturbances, thereby improving reproductive function, including oestrous expression and conception outcomes [20]. However, direct evidence demonstrating complete normalisation of progesterone profiles and reduction of early embryonic loss in goats remains limited.

Modifications to synchronisation protocols are also essential in endemic regions. Extending the duration of exogenous progesterone exposure can compensate for luteal insufficiency, while supplemental equine chorionic gonadotropin (eCG) supports follicular maturation and ovulation in does with compromised endocrine responsiveness [105,106,107]. Protocols that incorporate careful timing of device insertion relative to infection treatment ensure that the hypothalamic–pituitary–gonadal axis is adequately primed to respond, enhancing the success of fixed-time artificial insemination programs.

Nutritional and antioxidant support further mitigates molecular and oxidative damage to ovarian and testicular tissue [108]. Supplementation with selenium, vitamin E, and L-carnitine supports granulosa and luteal cell resilience, enhances steroidogenic enzyme activity, and reduces reactive oxygen species-mediated apoptosis [109,110,111,112]. Adequate energy and protein intake maintain metabolic homeostasis, ensuring substrate availability for cholesterol-dependent progesterone synthesis, thereby improving luteal competence and conception outcomes.

Integrated vector management complements these interventions by reducing exposure to tsetse and mechanical vectors. Approaches include strategic deployment of insecticide-treated targets and traps, application of pour-on insecticides to grazing animals, and vegetation management to limit vector habitats. By decreasing the intensity and frequency of parasitic challenge, these measures reduce systemic inflammatory burden and preserve reproductive function.

Genetic selection for reproductive resilience offers a longer-term strategy. Identifying and breeding WAD goats that combine trypanotolerance with preserved endocrine function and high reproductive efficiency can gradually shift herd composition toward animals that withstand parasitic challenge without substantial fertility loss. Integrating these traits into local breeding programs requires careful monitoring of oestrus cycles, conception rates, and kid survival alongside parasitological screening.

Cost–benefit frameworks are critical for smallholder adoption. Interventions must be economically feasible, considering the direct cost of drugs, synchronisation devices, and nutritional supplements relative to expected increases in kid crop, milk yield, and household income. Studies suggest that strategic treatment combined with modified synchronisation protocols can restore up to 70–80% of reproductive potential in previously subfertile herds, representing substantial returns on investment for rural farmers [29,35].

Ultimately, breaking the trypanotolerance paradox requires a holistic strategy that addresses both survival and fertility. By integrating parasitological, reproductive, nutritional, and vector control measures, smallholder farmers can maintain herd productivity, improve food security, and preserve livelihoods while leveraging the inherent resilience of WAD goats. A summary of integrated strategies to restore reproductive efficiency and mitigate the trypanotolerance paradox in WAD goats is provided in Table 7.

## 12. Impact of African Trypanosomiasis on Male Reproductive Function

Although reproductive dysfunction in trypanosome-infected goats has been predominantly studied in females, emerging evidence indicates significant impairment of male fertility parameters. Similar reproductive impairments have been documented in experimental infections of bucks with *Trypanosoma congolense* and *T. vivax*, where significant reductions in sperm motility, sperm concentration, and circulating testosterone were observed [103]. Chronic trypanosome infection in bucks is associated with reduced libido, decreased semen volume, lowered sperm concentration, impaired motility, and increased morphological abnormalities [105]. Histopathological evaluations reveal degeneration of seminiferous tubules, interstitial oedema, and disruption of spermatogenic cell layers, suggesting direct and indirect testicular injury.

**Figure 8:**
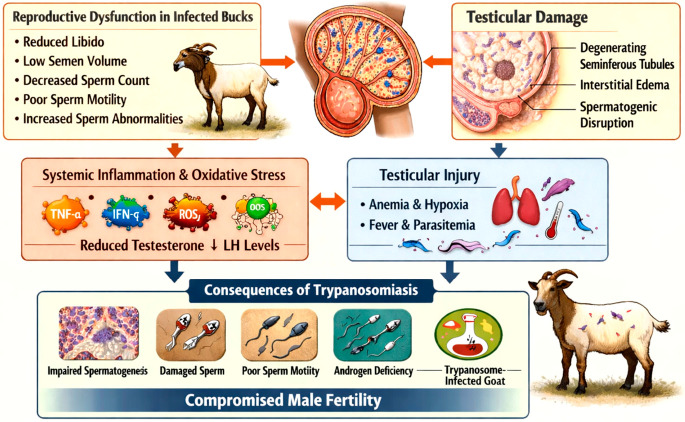
Pathophysiological mechanisms and reproductive consequences of African animal trypanosomiasis on male fertility in bucks.

Mechanistically, systemic inflammation and oxidative stress appear central to testicular dysfunction. Elevated pro-inflammatory cytokines, including TNF-α and IFN-γ, compromise Leydig cell steroidogenesis, leading to reduced testosterone synthesis [107,108,109]. Suppression of LH further exacerbates androgen deficiency, impairing spermatogenesis and libido [110,111,112]. Concurrently, ROS generation damages sperm membrane lipids and mitochondrial integrity, reducing motility and fertilising capacity [113,114,115,116]. Anaemia-induced hypoxia may additionally compromise testicular microcirculation, while fever associated with acute parasitaemia can disrupt thermoregulation critical for spermatogenic efficiency. In trypanotolerant breeds, survival does not necessarily equate to preserved reproductive competence, and subclinical endocrine suppression may persist despite controlled parasitaemia. These findings underscore that the reproductive cost of trypanotolerance extends to both sexes, reinforcing the need for integrated fertility monitoring in endemic systems. To complement the discussion on female reproductive impairment, the mechanistic pathways through which African animal trypanosomiasis compromises male reproductive function are illustrated in Figure 8. The figure summarises the cascade linking chronic trypanosome infection with systemic inflammation, oxidative stress, endocrine disruption, and testicular tissue damage, culminating in impaired spermatogenesis, reduced semen quality, and compromised fertility in infected bucks.

## 13. Future Research and Policy Directions

Despite extensive research on African animal trypanosomiasis, its reproductive impacts in WAD goats remain insufficiently explored, with emphasis historically placed on survival rather than fertility. Future studies should prioritise mechanistic investigations linking parasitaemia, cytokine responses, and oxidative stress to ovarian steroidogenesis, luteal function, and follicular dynamics. Longitudinal field studies integrating molecular diagnostics, such as PCR targeting variant surface glycoproteins, with endocrine monitoring will improve detection of subfertility and early embryonic loss. Incorporating reproductive indicators—progesterone profiling, oestrous tracking, and pregnancy outcomes—into surveillance systems is essential for assessing herd productivity.

Research into adaptive breeding strategies that balance trypanotolerance with reproductive efficiency is urgently needed, particularly through the identification of genetic markers of ovarian resilience. Policy frameworks should address the economic burden of subfertility by promoting parasite screening, strategic treatment, improved nutrition, and modified synchronisation protocols. Integrated vector management and multi-sectorial collaboration are critical, with a One Health approach ensuring sustainable productivity and livelihood security in endemic regions.

### Limitation of the Study

The relatively small number of goat-specific reproductive studies that met the PRISMA eligibility criteria warranted cautious contextualisation of some mechanistic interpretations using comparative reproductive and immunopathological literature from related animal models, other than WAD goats. Despite this limitation, the study provides a comprehensive and evidence-informed synthesis of the reproductive, physiopathological, and One Health implications of caprine trypanosomosis, particularly in WAD goats.

## 14. Conclusions

Animal trypanosomiasis remains a major but under-recognised constraint to reproductive performance and productivity in WAD goats. Evidence from the reviewed studies indicates that trypanosome infection disrupts reproductive function through interconnected mechanisms involving anaemia, endocrine imbalance, chronic inflammation, oxidative stress, and metabolic dysfunction. Available evidence suggests that *T. brucei* may more frequently be associated with ovarian dysfunction and embryonic loss, whereas *T. congolense* has been linked in some studies to uterine pathology and mid-to-late gestational abortions. These reproductive impairments reduce fertility, kid survival, herd growth, and overall livestock productivity despite the relative trypanotolerance of WAD goats. Integrated control strategies combining vector control, surveillance, targeted chemotherapy, nutritional support, and selective breeding are therefore essential for sustainable disease mitigation and improved reproductive resilience.

## Figures and Tables

**Table 1:** Characteristics and key findings of the 14 studies included in the PRISMA-derived systematic synthesis on caprine trypanosomosis and reproductive dysfunction.

S/n	Author(s)	Country	Study Design	Goat Breed/Population	Trypanosome Species	Main Reproductive Outcome(s)	Mechanistic/Pathological Findings	Key Contribution to Review
1	Mutayoba et al. (1988) [18]	Tanzania	Experimental infection	Female goats	*T. congolense*	Reduced fertility, altered cyclicity	Decreased progesterone and estradiol concentrations	Provided endocrine evidence linking trypanosomosis to ovarian dysfunction
2	Faye et al. (2004) [19]	Senegal	Experimental study	West African Dwarf goats	*T. congolense*	Reduced reproductive performance	Anaemia and metabolic stress associated with reproductive decline	Demonstrated production and reproductive impacts
3	Adamu et al. (2009) [20]	Nigeria	Experimental pathology study	Male and female goats	*T. brucei*	Gonadal dysfunction	Testicular degeneration and ovarian lesions	Provided histopathological evidence of reproductive tissue injury
4	Gutierrez et al. (2006) [21]	Multi-country Africa	Review	Goats	Multiple species	General reproductive impairment	Chronic inflammatory and systemic effects	Contextualised caprine trypanosomosis burden
5	Faye et al. (2005) [22]	Senegal	Experimental infection	WAD goats	*T. congolense*	Reduced milk production and productivity	Biochemical alterations linked to chronic infection	Demonstrated economic and physiological consequences
6	d’Ieteren et al. (1998) [23]	Africa	Review	Indigenous ruminants	Multiple species	Trypanotolerance traits	Genetic and adaptive resistance mechanisms	Supported discussion on breed resilience
7	Yaro et al. (2016) [6]	Burkina Faso	Review	Livestock populations	Multiple species	Production and reproductive losses	Trypanotolerance and disease adaptation	Supported sustainable control discussions
8	Desquesnes et al. (2013) [24]	Global	Review	Multiple livestock species	*T. evansi*	Infertility and abortion	Systemic inflammatory and endocrine disruption	Supported comparative reproductive pathogenesis
9	Gitonga et al. (2017) [7]	Kenya	Experimental infection	Small ruminants	*T. congolense*/*T. brucei*	Variable reproductive outcomes	Strain virulence differences	Supported pathogenic variability discussion
10	Van den Bossche et al. (2011) [25]	Southern Africa	Experimental epidemiology	Ruminants	*T. congolense*	Disease severity variation	Virulence-associated pathology	Supported disease severity interpretation
11	Hassan-Kadle et al. (2020) [26]	Somalia	Cross-sectional study	Sheep and goats	Multiple species	Reproductive health concerns	Molecular detection of circulating trypanosomes	Supported epidemiological burden
12	Maganga et al. (2020) [27]	Gabon	Molecular epidemiology	Sheep and goats	Multiple species	Infection prevalence	Trypanosome diversity	Supported geographic distribution evidence
13	Daniel et al. (1994) [28]	Nigeria	Field prevalence study	Goats and sheep	Multiple species	Reduced productivity	Endemic exposure patterns	Supported endemicity discussions
14	Ohaeri (2010) [29]	Nigeria	Cross-sectional survey	Ruminants	Multiple species	General production losses	High prevalence in communal systems	Supported field-level disease burden

**Table 2:** Summary of species-specific trypanosome-induced reproductive and endocrine disruptions in goats.

Trypanosome Species	Host Category	Reproductive Parameters Affected	Endocrine Changes	Molecular/Cellular Mechanisms	Clinical/Field Manifestation	References
*T. brucei brucei*	Does	Irregular oestrous cycles, embryonic loss	Decreased progesterone and LH	HPG axis disruption; inflammatory cytokines	Silent heat, infertility	[18,19,20]
*T. brucei brucei*	Does	Follicular atresia, luteal degeneration	Decreased progesterone	Oxidative stress; granulosa apoptosis	Anovulation	[18,19]
*T. congolense*	Does	Reduced conception, anoestrus	Decreased progesterone and oestradiol	Hypothalamic dysfunction; impaired GnRH release	Delayed breeding	[18,19,20]
*T. congolense*	Does	Suppressed LH surge	Decreased LH pulsatility	Pituitary–hypothalamic axis disruption	Irregular cycles	[18,21]
*T. vivax*	Bucks	Reduced sperm quality, infertility	Decreased testosterone	Testicular degeneration; inflammation	Poor semen quality	[22]
*T. vivax*	Does	Anoestrus, infertility	Altered progesterone	Ovarian dysfunction	Failure to conceive	[23,24]
*T. vivax*	Pregnant does	Abortion, foetal loss	Hormonal imbalance	Placental damage; transplacental infection	Pregnancy loss	[25]
*T. evansi*	Bucks	Reduced fertility, orchitis	Decreased testosterone	Testicular atrophy; inflammatory infiltration	Aspermatogenesis	[22]
Mixed infections	Herd	Subfertility, reduced productivity	Endocrine dysregulation	Chronic inflammation; anaemia	Reduced herd growth	[26,27]
Chronic infection (trypanotolerant goats)	Herd	Delayed puberty, low fertility	Altered HPG axis	Energy diversion; immune activation	Reduced productivity	[19]

**Table 3:** African trypanosome species, transmission mode, and relative reproductive impacts in WAD goats.

Trypanosome Species	Transmission Mode	Typical Parasitaemia	Hormonal Disruption (Progesterone/LH/FSH)	Reproductive Outcome	References
*T. congolense*	Tsetse	Chronic, low-moderate	decreased progesterone, decreased LH	Silent oestrus, early embryonic loss, abortion	[20]
*T. vivax*	Tsetse fly/Mechanical	Acute or chronic	Altered oestradiol, luteal insufficiency	Reduced conception, abortion	[33]
*T. brucei brucei*	Tsetse fly	Low-moderate	Oxidative stress, decreased LH	Anovulation, follicular atresia	[34]
*T. evansi*	Mechanical	Variable, often chronic	Decreased progesterone, ↑ inflammatory cytokines	Early embryonic loss, abortion	[35]

**Table 4:** Epidemiological distribution and prevalence of *Trypanosoma* spp. infections in goats and sheep across African endemic regions: evidence from parasitological and molecular studies.

Countries	Host (n)	Prevalence	Diagnostic Method	Species Identified/Reported	References
Somalia	Goats (206)	17.5%	PCR	*T. evansi*, *T. godfreyi*, *T. vivax*, *T. brucei*, *T. simiae*, *T. congolense*	[37]
Somalia	Sheep (206)	8.3%	PCR	Same spp. reported	[37]
Gabon	Goats (118)	16.1%	PCR	*T. vivax*, *T. simiae*, *T. simiae Tsavo*, *T. congolense*, *T. brucei*	[38]
Gabon	Sheep (100)	21.0%	PCR	*T. simiae*, *T. theileri*	[38]
Nigeria	Goats (357)	5.0%	Microscopy	*T. vivax*, *T. congolense*, *T. brucei*	[40]
Nigeria	Sheep (258)	7.4%	Microscopy	*T. vivax*, *T. congolense*, *T. brucei*	[40]
Nigeria	Goats (81)	1.2%	Microscopy	*T. vivax*, *T. congolense*	[41]
Nigeria	Sheep (67)	1.1%	Microscopy	*T. vivax*, *T. congolense*	[41]
Nigeria	Goats (98)	1.64%	Microscopy	*T. vivax*, *T. congolense*, *T. brucei*	[42]
Nigeria	Sheep (64)	3.02%	Microscopy	*T. vivax*, *T. congolense*, *T. brucei*	[42]
Uganda	Goats (82)	13.88%	DNA methods	*T. brucei* most common	[39]
Uganda	Sheep (64)	8.51%	DNA methods	*T. brucei* most common	[39]

**Table 5:** Reproductive and productivity impacts of African animal trypanosomiasis in West African Dwarf goats.

Reproductive Parameter	Healthy WAD Goats	Trypanosome-Infected Goats	Reported Impact	Mechanistic Interpretation	References
Conception rate	70–85%	40–65%	Markedly reduced conception efficiency	Luteal insufficiency; progesterone suppression; impaired ovulation	[18,20]
Kidding interval	7–9 months	12–16 months	Prolonged inter-kidding interval	Delayed return to oestrus; endocrine disruption of HPG axis	[20]
Abortion rate	<5%	10–25%	Increased foetal loss and pregnancy wastage	Placental insufficiency; systemic inflammation; fever-induced embryonic death	[20,63]
Kid birth weight	1.8–2.5 kg	1.2–1.8 kg	Reduced foetal growth (intrauterine growth restriction)	Maternal anaemia; reduced nutrient transfer; placental dysfunction	[20,33]
Neonatal mortality	5–10%	15–35%	Increased pre-weaning mortality	Weak neonates; poor colostrum quality; maternal illness	[33]
Annual kid crop	1.5–2.0 kids/doe/year	0.8–1.2 kids/doe/year	Substantial reduction in herd productivity	Combined effects of infertility, abortion, and kid mortality	[20]

Values represent ranges reported in experimental infections and field-based epidemiological studies across sub-Saharan Africa. Mechanistic interpretations are supported by controlled experimental studies and comparative ruminant models where necessary.

**Table 6:** Molecular mechanisms of ovarian dysfunction in trypanosome-infected WAD goats.

Mechanism	Molecule/Gene	Target Tissue	Reproductive Effect	Reference
Immune-mediated suppression	TNF-α, IL-1β, IFN-γ	Hypothalamus/Pituitary/Ovary	decreased GnRH, LH, FSH; luteal insufficiency	[97]
Steroidogenic enzyme inhibition	StAR, CYP11A1, HSD3B1, CYP19A1	Granulosa & luteal cells	decreased Progesterone, decreased Oestradiol	[20]
Oxidative stress	ROS, NO	Granulosa & theca cells	Follicular apoptosis, atresia	[34]
Apoptotic signalling	Bax ↑/Bcl-2 decreased, Caspase-3/9	Granulosa & luteal cells	Anovulation, follicular loss	[34]
Premature luteolysis	PGF2α ↑, COX-2	Corpus luteum	Shortened luteal phase, early embryonic loss	[20]

**Table 7:** Integrated intervention strategies to mitigate trypanosome-induced reproductive failure in WAD goats.

Strategy	Mechanistic Target	Expected Reproductive Outcome	Field Implementation	Reference
Pre-breeding parasite screening & trypanocidal therapy	Reduce parasitaemia, inflammation	↑ Progesterone, ↑ conception rate	PCR detection followed by treatment prior to breeding	[20,100]
Modified progesterone synchronisation	Compensate for luteal insufficiency	Synchronised oestrus, ↑ ovulation	Extended CIDR/eCG protocols	[103]
Nutritional & antioxidant support	Reduce oxidative stress	decreased follicular apoptosis, ↑ luteal function	Selenium, Vitamin E, L-carnitine supplementation	[97]
Vector control	Reduce infection pressure	Decreased inflammatory burden, improved reproductive efficiency	Insecticide-treated targets, pour-ons, habitat management	[29]
Genetic selection for reproductive resilience	Enhance endocrine robustness	Higher conception rate & kid survival	Breeding programs targeting ovarian resilience	[61]

## Data Availability

The original contributions presented in this study are included in the article/Supplementary Material. Further inquiries can be directed to the corresponding authors.

## Supplementary Materials

The following supporting information can be downloaded at: https://www.mdpi.com/article/10.3390/vetsci13060535/s1, Table S1: Simplified methodological quality and risk-of-bias assessment of included studies (n = 14); Table S2: PRISMA 2020 Checklist for Reporting Systematic Reviews.

## Abbreviations

The following abbreviations are used in this manuscript:

AAT	African Animal Trypanosomiasis
WAD	West African Dwarf (goats)
HPG	Hypothalamic–Pituitary–Gonadal
GnRH	Gonadotropin-Releasing Hormone
LH	Luteinising Hormone
FSH	Follicle-Stimulating Hormone
ROS	Reactive Oxygen Species
TNF-α	Tumour Necrosis Factor-alpha
IFN-γ	Interferon-gamma
IL-1β	Interleukin-1 beta
StAR	Steroidogenic Acute Regulatory protein
CYP11A1	Cytochrome P450 side-chain cleavage enzyme
HSD3B1	3β-Hydroxysteroid Dehydrogenase
CYP19A1	Aromatase
PGF2α	Prostaglandin F2-alpha
COX-2	Cyclooxygenase-2
CIDR	Controlled Internal Drug Release (progesterone device)
eCG	Equine Chorionic Gonadotropin
PCR	Polymerase Chain Reaction
VSG	Variant Surface Glycoprotein
