# Supplementary material for: Reproductive Impacts of African Animal Trypanosomiasis in West African Dwarf Goats—Mechanistic Insights into Trypanotolerance Survival–Fertility Trade-Off: A Systematic Review
Source: Vet Sci. 2026 May 29;13(6):535. doi: 10.3390/vetsci13060535 (PMC13308315; doi:10.3390/vetsci13060535)
Supplement: Supplementary file 1 [file vetsci-13-00535-s001.zip › vetsci-4281773-supplementary.pdf]

Table S1. Simplified methodological quality and risk-of-bias assessment of included studies (n = 14)

S/N	Study	Country	Study design	Sample size (n)	Methodological clarity	Overall risk of bias
1	Mutayoba et al.	Tanzania	Experimental	20–100	Moderate	High
2	Faye et al.	Senegal	Experimental	20–100	Moderate	Moderate
3	Adamu et al.	Nigeria	Experimental	<20	Moderate	High
4	Gutierrez et al.	Multi-country	Review	N/A	Moderate	Moderate
5	Faye et al.	Senegal	Experimental	20–100	Moderate	Moderate
6	d'Ieteren et al.	Africa	Review	N/A	High	Low
7	Yaro et al.	Burkina Faso	Review	N/A	High	Low
8	Desquesnes et al.	Global	Review	N/A	High	Low
9	Gitonga et al.	Kenya	Experimental	<20	Moderate	Moderate
10	Van den Bossche et al.	Southern Africa	Experimental	20–100	Moderate	Moderate
11	Hassan-Kadle et al.	Somalia	Cross-sectional	100+	Moderate	Moderate
12	Maganga et al.	Gabon	Molecular epidemiology	20–100	High	Low
13	Daniel et al.	Nigeria	Field study	100+	Moderate	Moderate
14	Ohaeri	Nigeria	Cross-sectional	100+	Moderate	Moderate

Table S2. PRISMA 2020 Checklist for Reporting Systematic Reviews

Section	Item No.	Checklist Item	Reported (Yes/No)	Location in Manuscript
Title	1	Identify the report as a systematic review	Yes	Title
Abstract	2	Structured summary including objectives, methods, results, conclusions	Yes	Simple summary and Abstract sections
Introduction	3	Rationale for review	Yes	Introduction
Introduction	4	Objectives clearly stated	Yes	Introduction
Methods	5	Eligibility criteria defined	Yes	Methods
Methods	6	Information sources described	Yes	Methods
Methods	7	Search strategy reported	Yes	Methods
Methods	8	Selection process described	Yes	Methods
Methods	9	Data collection process described	Yes	Methods
Methods	10	Data items defined	Yes	Methods
Methods	11	Risk of bias assessment	Partial (simplified)	Methods
Methods	12	Effect measures (if applicable)	Not applicable	Methods
Methods	13	Synthesis methods described	Yes	Methods
Methods	14	Reporting bias assessment	No	Not performed
Methods	15	Certainty assessment (e.g. GRADE)	No	Not performed
Results	16	Study selection process (PRISMA flow)	Yes	Results
Results	17	Study characteristics reported	Yes	Tables 1 to 7
Results	18	Risk of bias within studies	Yes (simplified)	Tables 1 to 7
Results	19	Results of individual studies	Yes	Results section
Results	20	Synthesis of results	Yes	Results/Discussion
Discussion	21	Summary of evidence	Yes	Discussion
Discussion	22	Limitations	Yes	Discussion
Discussion	23	Interpretation	Yes	Discussion
Other	24	Funding	No	End section
Other	25	Conflicts of interest	No	End section
